# Clinical Observations Concerning the Heart in Syphilis

**Published:** 1913-09

**Authors:** 


### ABSTRACTS.

Clinical Observations Concerning the Heart in Syphilis. Harlow Brooks, *Interstate Med. Jour.*, 1913, xx, 507. According to Brooks' statistics, involvement of the heart is responsible for death, or is a very important contributing factor to it, in 70 per cent. of the mortalities in syphilis. In a systematic study of fifty consecutive autopsies in individuals who had succumbed during the tertiary or quartenary stages of syphilis, grave heart lesions were found in from 50 to 80 per cent. In syphilis the heart may become involved very early in the secondary stage of the disease, although the majority of cases probably develop rather late; perhaps twenty or thirty years after the initial lesion. A clear history of luetic infection is obtained in a relatively few cases of syphilis of the heart; so that in a series of cases in which the diagnosis was made or verified at autopsy there was no obtainable history in 70 per cent. The majority of the cases present themselves complaining of dyspnea on exertion, cardiac pain, palpitation, throbbing arteries and weakness. Myocarditis, endocarditis, or aortitis are made out on physical examination. In order of frequency the lesions found by Brooks have been (1) associated mitral and aerotic disease, (2) aerotic endocarditis, (3) irregular involvement of the other valves. Although mitral disease is fairly frequent in syphilis, that infection shows a definite affinity to attack the aortic valves and the conus arteriosus. When a suitable history cannot be obtained from the patient it is reasonable to assume that an aortitis is caused by lues until other explanatory data can be obtained. The Wasserman reaction should be done in all cases. The disease is progressive and although definite valvular signs and symptoms may be present the factor of myocardial involvement becomes daily and progressively more dominant. A positive Wasserman reaction obtained by a competent observer after the employment of the older Wasserman technique is incorrect in very few instances. There are some cases in which the Wasserman reaction has become positive after antisyphilitic treatment has been instituted. The prognosis should depend upon the results of the antisyphilitic treatment. Sometimes endocardial murmurs disappear completely. The primary treatment should be along cordiac lines. Later, salversan intravenously (0.3 gm.) followed by mercury intramuscularly or by inunction or by the mouth. The salvarsan should be repeated in a week or a month. After all symptoms have disappeared one month should be allowed to pass without treatment and then the Wasserman reaction should be tried again. If it is negative, treatment may be discontinued for another three months and the Wasserman reaction again tried. The author is of the opinion that the iodides have little specific action against the syphilitic virus; yet he uses them for their effect in promoting the absorption of deposits and exudates.

